# Clinical analysis of tracheobronchial foreign body aspiration in children: a focus on external and intrinsic factors

**DOI:** 10.1186/s12893-021-01089-3

**Published:** 2021-03-03

**Authors:** Weigang Gan, Ning Xiao, Yiyuan Feng, Danmei Zhou, Juanjuan Hu, Shixi Liu, Jian Zou

**Affiliations:** 1 Department of Otorhinolaryngology–Head and Neck Surgery, West China Hospital, West China School of Medicine, Sichuan University, Chengdu, Sichuan People’s Republic of China; 2 Health Management Center, Sichuan Provincial People’s Hospital, Chengdu, Sichuan People’s Republic of China; 3 Department of Otorhinolaryngology–Head and Neck Surgery, Qianwei People’s Hospital, Sichuan 614400 Leshan, People’s Republic of China

**Keywords:** Tracheobronchial foreign body aspiration, Rigid bronchoscopy, Guardians, External factors, Intrinsic factors

## Abstract

**Background:**

Tracheobronchial foreign body aspiration (TFBA) is a critical disease in children and is extremely dangerous, even life-threatening. The factors affecting the occurrence and prognosis of TFBA are complex. The purpose of this study is to examine the external and intrinsic factors affecting clinical features of TFBA in West China and propose potential effective intervention measures.

**Methods:**

We retrospectively analyzed the clinical data of pediatric patients diagnosed with TFBA with foreign bodies (FBs) removed by rigid bronchoscopy under general anesthesia at the otolaryngology department from December 2017 to November 2018. The data included age, sex, clinical symptoms, type and location of FB, guardians, prehospital duration and residence of these pediatric patients.

**Results:**

The ratio of males (72) to females (53) was 1.4:1. Children aged from 1 to 3 years accounted for 76% (95/125) of patients. Cough, continuous fever and dyspnea were the primary symptoms. The right primary bronchus was the most common location of FB detection by rigid bronchoscopy (67 cases, 53.6%). Organic FBs were most common in our study. Guardians of patients significantly differed in the rural (parents 16, grandparents 31) and urban (parents 52, grandparents 26) groups (*χ*^2^ = 12.583, *p* = 0.000). More children in the rural group than in the urban group had a treatment delay longer than 72 h. More children in the group with no history of FB aspiration (12, 25%) than in the group with prior FB aspiration had a treatment delay longer than 72 h.

**Conclusion:**

Pediatric TFBA is a common emergency in otolaryngology. Age, sex, tracheobronchial anatomy and other physiological elements were defined as intrinsic factors, while guardians, residence, FB species and prehospital time were defined as external factors of TFBA. External and intrinsic factors both influence the occurrence and progression of TFBA. It is extremely important to take effective measures to control external factors, which can decrease morbidity and mortality.

## Background

Pediatric tracheobronchial foreign body aspiration (TFBA) is the most hazardous and most common emergency among respiratory tract diseases; it not only initiates infection but also causes obstructive asphyxia or spasmodic asphyxia [1]. The 2008 statistics from the National Safety Council (NSC) revealed that over 17 thousand pediatric patients in the United States who were younger than 14 years presented to the emergency department, and approximately 220 patients eventually died due to TFBA in that year alone [2]. Hence, it is extremely important for patients to be examined by an emergency medicine specialist in a timely manner and receive professional treatment if they are suffering from TFBA. However, when and whether children are taken to the hospital depends on multiple factors related to their guardians, such as economic status, geographical location, education degree and disease cognition. West China is characterized by mountainous areas that have less developed infrastructure and economic conditions than East China. We analyzed the clinical data to determine the external and intrinsic factors that affect the clinical features of TFBA in this area. The external factors were related to the living environment, and the intrinsic factors referred to physiological factors such as age and tracheobronchial anatomy.

## Methods

This study was approved by the Institutional Review Board of the West China Hospital and performed at the West China Hospital National Diagnosis and Treatment Center of West China for Emergency and Critical Disease (NO. 2017–448). We retrospectively analyzed the clinical data of pediatric patients diagnosed with TFBA with foreign bodies (FBs) removed by rigid bronchoscopy under general anesthesia at the otolaryngology department from December 2017 to November 2018. Owing to the development of the respiratory tract and physiological function, older children manifest more potential safety risks than infants do. Thus, we enrolled children younger than 7 years who had not entered the first grade of primary school and who had been diagnosed with TFBA by computed tomography (CT) scan, auscultation, symptoms or history of FB aspiration, which was further verified by rigid bronchoscopy. The clinical data collected consisted of demographics (age distribution, sex), residence (rural, urban), guardians (parents, grandparents), aspiration history (witnessed or not, length of time before treatment), symptoms, bronchoscopic findings and types of FBs. Statistically, qualitative data or proportions were analyzed by Fisher's exact test or Pearson's *χ*^2^ test. In our study, qualitative data included the sample quantity in subgroups divided according to demographic factors (age distribution, sex), residence (rural, urban), guardians (parents, grandparents), aspiration history (witnessed or not, length of time before treatment) and types of FBs.

SPSS software (version 22.0, IBM, Chicago, USA) was used to conduct the statistical analysis. A value of *p* < 0.05 (two tailed) was considered statistically significant.

## Results

### Patient demographics

In total, 125 pediatric patients diagnosed with TFBA and treated by rigid bronchoscopy under general anesthesia at the otolaryngology department of the West China Hospital from December 2017 to November 2018 were enrolled in our study. The ratio of males (72) to females (53) was 1.4:1. The age distribution ranged from 7 months to 6 years, with a mean age of 2.02 ± 1.13 years; children younger than 1 year accounted for 11.2% (14/125), those aged 1 to 3 years accounted for 76% (95/125), and those older than 3 years accounted for 12.8% (16/125) of patients (Table 1). Among children younger than 1 year old, the patients included 7 males and 7 females, and the FBs included 3 cases involving peanuts, 8 cases involving seeds, 2 cases involving sarcocarps, and 1 case involving rice. The locations included the right primary bronchus in 7 cases, left primary bronchus in 6 cases, and trachea in 1 case.

**Table 1:** Demographics and clinical characteristics of the study cohorts

	Quantity	
	No	Proportion (%)
Gender		
Male	72	57.6
Female	53	42.4
Age (month)		
≤ 12	14	11.2
13–36	95	76.0
> 36	16	12.8
Prehospital time (h)		
< 24	72	57.6
24–72	36	28.8
≥ 72	17	13.6
Types of foreign bodies		
Peanuts	51	40.8
Sunflower seeds	32	25.6
Walnut seeds	23	18.4
Rice and noodles	13	10.4
Sarcocarp	4	3.2
Metal	1	0.8
Plastics	1	0.8
Foreign body locations		
Left primary bronchus	49	39.2
Right primary bronchus	67	53.6
Trachea	9	7.2
Clinical symptoms		
Coughing	114	91.2
Heaving	72	57.6
Fever	56	44.8
Dyspnea	48	38.4
Wheezing	46	36.8
Residences		
Rural	47	37.6
Urban	78	62.4
Guardians		
Parents	68	54.4
Grand parents	57	45.6

### Clinical manifestations

Regarding clinical symptoms, 114 patients suffered from cough as a major symptom (91.2%), 72 patients suffered from heaving as a major discomfort (57.6%), 56 patients suffered from continuous fever (44.8%), 48 children had dyspnea as the most distressing symptom (38.4%), and 46 pediatric patients exhibited obvious wheezing (36.8%) (Table 1). Regarding the location of the FBs, the right primary bronchus was the most common position involved, with FBs detected by rigid bronchoscopy in 67 cases (53.6%), followed by the left primary bronchus (49/125, 39.2%) and trachea (9/125, 7.2%). The FB was successfully removed in 125 cases, and the location of the FB was consistent with the preoperative imaging examination (Table 1). Position variance of FBs from the right bronchus to the left bronchus was observed in 3 cases during the operation.

Types of foreign bodies.

Diverse types of FBs were documented in our study. In decreasing order of occurrence, the FBs included peanuts (51, 40.8%), sunflower seeds (32, 25.6%, 18 cases with only the shell and 14 cases with both the shell and the seed), walnuts (23, 18.4%), rice or noodles (13, 10.4%), sarcocarps (4, 3.2%), and metal and plastics (0.8%) (Table 2).

**Table 2:** Composition ratio of the types of foreign bodies between urban and rural children (n,%)

	Urban		Rural		Count	
Types of fbs	n	%	n	%	n	%
Peanuts	31	39.8	20	42.6	51	40.8
Sunflower seeds	20	25.6	12	25.6	32	25.6
Walnuts	15	19.2	8	17.0	23	18.4
Rice and noodle	8	10.3	5	10.6	13	10.4
Sarcocarp	3	3.8	1	2.1	4	3.2
Metal	1	1.3	0	0	1	0.8
Plastics	0	0	1	2.1	1	0.8
Count	78	100	47	100	125	100

*χ*^2^ = 2.580, *p* = 0.946 > 0.05

### Prehospital elements

In terms of residence, 47 patients lived in rural areas (37.6%), while 78 patients lived in urban areas (62.4%). Regarding guardians, 68 children were cared for daily by parents (54.4%); however, 57 were cared for by grandparents (45.6%). It was inquired of all patients whether the FB had been inhaled while eating or playing with the object in the child’s mouth. The longest prehospital time after FB aspiration was 17 days, and the shortest prehospital time was 0.5 h; 72 patients (57.6%) were brought to the hospital within one day, 36 patients were brought to the hospital between 1 and 3 days (28.8%), and 17 patients were brought to the hospital after more than 3 days (13.6%) (Table 1).

Among urban and rural children, the most common FBs were peanuts (31 vs 20), sunflower seeds (20 vs 12), and walnuts (15 vs 8), with no significant difference between groups (*χ*^2^ = 2.580, *p* = 0.946 > 0.05). The sex ratio of the rural (40 females, 19 males) and urban (32 females, 34 males) groups was not significantly different (*χ*^2^ = 0.934, *p* = 0.362). The guardians of patients in the rural (parents 16, grandparents 31) and urban (parents 52, grandparents 26) groups were significantly different (*χ*^2^ = 12.583, *p* = 0.000). The prehospital times after FB aspiration in the rural (< 24 h, 32; 24–72 h, 7; ≥ 72 h, 8) and urban (< 24 h, 40; 24–72 h, 29; ≥ 72 h, 9) groups were significantly different (*χ*^2^ = 7.144, *p* = 0.028). Although most patients in both groups were taken to the doctor within 24 h, more children were treated from 24 to 72 h in the urban group than in the rural group (29/78 vs 7/47), and fewer children were treated after more than 72 h in the urban group than in the rural group (9/78 vs 8/47) (Table 3).

**Table 3:** Differences in gender, guardians and prehospital time between the two groups (n)

	Gender		Caregivers		Prehospital time (h)		
Residence	Female	Male	Parents	Grandparents	< 24	24–72	≥ 72
Urban	32	19	52	26	40	29	9
Rural	40	34	16	31	32	7	8
Count	72	53	68	57	72	36	17
*χ*^2^	0.934		12.583		7.144		
*P*	0.362		0.000		0.028^※^		

When pediatric patients were divided into two groups according to prior FB aspiration, a significant difference was observed between the two groups in the time until a doctor was consulted (*χ*^2^ = 11.573, *p* = 0.003). Although most patients in both groups were treated at a pediatric clinic or emergency room within 24 h, more children in the group without prior FB aspiration (12, 25%) were treated after more than 72 h than in the group with previous FB aspiration (5, 6.5%) (Table 4). After 1 year of follow-up, pediatric patients with TFBA in our group had no complications, including pulmonary dysfunction.

**Table 4:** Prehospital time after FB aspiration between two groups (n, %)

Prehospital time(h)	With history		Without history		Count	
< 24	52	67.5	20	41.7	72	57.6
24–72	20	26.0	16	33.3	36	28.8
≥ 72	5	6.5	12	25.0	17	13.6
Count	77	100	48	100	125	100

*χ*^2^ = 11.573, *p* = 0.003 < 0.05, History = history of FB aspiration

## Discussion

Pediatric TFBA refers to children who suffer from aspiration of a variety of objects by mistake while crying, smiling or yelling. It is very important to consult specialists for professional aid immediately after aspiration occurs or to avoid it from the very beginning. In our opinion, the occurrence of TFBA is influenced by several external and intrinsic factors. Here, we aim to discuss these factors and search for potentially effective measures corresponding to these factors.

Physiological elements of children were defined as intrinsic factors, including age, sex, tracheobronchial anatomy and symptoms. Accordingly, elements irrelevant to children were defined as external factors, including guardians, residence, type of FB and prehospital time.

Owing to both the laryngeal protective function and masticatory function development, TFBA usually occurs in young children, especially those less than 3 years old [3]. In our study, the age distribution of pediatric patients was mostly from 1 to 3 years, coinciding with Arias’s findings from the National Center for Health Statistics [4]. In addition, there were 72 boys and 53 girls enrolled in our study, and the male–female ratio was 1.36, similar to that in Maha’s study [5]. As is well known, infants attempt to explore the world with their tongues and mouths, and boys typically show stronger curiosity and relative hyperactivity compared to girls in daily life. Therefore, we must repeatedly emphasize to guardians that infants should not be permitted to eat nuts. It is best to put nuts and tiny objects out of children’s sight and reach.

In regard to bronchial anatomy, which affects the movement track of FBs, the right primary bronchus is steeper and wider than the left side [6]. As a result, the occurrence rate of right bronchial FBs is higher than that of other types [7]. FBs in our study were mostly located in the right bronchus, followed by the left bronchus and trachea. Tracheal FBs are fatal, despite a low incidence of morbidity [8]. In some cases, FBs migrated after violent coughing or patting of the back. Position variance of FBs from the right bronchus to the left bronchus was observed in 3 patients during the operation.

Cough, heaving, fever, and dyspnea were the four symptoms of highest incidence in our patients. The symptoms mainly depended upon the prehospital duration and the type of FB. Tan et al. concluded that nuts and seeds, with some geographic and seasonal variations, were the most common FBs [9]. In our group, most of the FBs were organic objects, such as peanuts, sunflower seeds and walnuts, which produce unsaturated fatty acid and thus promote inflammation of the airway mucosa.

Most of western China is mountainous, and rural and urban districts are different in terms of infrastructure and economic conditions. We regard the regional difference as an external factor of TFBA. When the patients were divided into two groups according to their residence, we found a significant difference in the guardians and medical history time but not in the sex of patients or the composition of the types of FBs.

Parents of pediatric patients with TFBA are younger than their grandparents, and the parents are also generally more educated than the grandparents. Guardians mostly included grandparents in rural areas because some parents in rural areas migrated to urban districts to search for employment, leaving children to be cared for by the grandparents. However, some parents also took children with them into urban districts, leading to fewer children living in villages and fewer patients with TFBA from rural areas. In our study, more pediatric patients were from urban areas, and more ill children were cared for by grandparents in rural areas. Therefore, in addition to providing information for parents, we must also educate grandparents, especially in rural families.

Administering TFBA therapy as soon as possible upon arrival at the hospital is key to timely diagnosis and surgery [8]. However, Foltran et al. found that approximately 40% of patients received delayed diagnosis and treatment [10], and more than 72 h of prehospital time was considered to potentially increase the risk of complications such as pneumonia, atelectasis, pneumorrhachis, pneumothorax, subcutaneous emphysema and pneumomediastinum [11].

When we focused on the medical history time, significant differences were found between children with or without a history of FB aspiration. A total of 67.5% of pediatric patients with a history of FB aspiration were immediately dealt within 24 h, while 58.3% of patients who had no explicit medical history were treated after 24 h. Meanwhile, children living in different districts also showed differences in prehospital time. Although most patients in both groups were taken to the doctor within 24 h, more children were not treated for over 72 h in the rural group than in the urban group. Guardians in rural areas were mostly grandparents, who possibly spend inadequate time caring for children and showed a lack of understanding about cognition of TFBA. Along with distance from medical centers or competent hospitals, the type of guardian was also associated with delayed diagnosis or therapy.

As a result, it is crucial for the guardian to be aware of the danger of TFBA, which could reduce its incidence and prevent medical delay.

## Conclusion

Pediatric TFBA is a common emergency in otolaryngology. Age, sex, tracheobronchial anatomy and other physiological elements were defined as intrinsic factors, while guardians, residence, type of FB and prehospital time were defined as external factors of TFBA. External and intrinsic factors both influence the occurrence and progression of TFBA. It is very important to take effective measures to control external factors, which can decrease morbidity and mortality.

## Data Availability

The dataset used and/or analyzed during the current study is available from the corresponding author on reasonable request.

## Abbreviations

FB: Foreign body
TFBA: Tracheobronchial foreign body aspiration
NSC: National Safety Council
CT: Computed tomography
